# The “Swing-Door” Regrafting of Donor Site: An Alternative Method for Split-Thickness Skin Graft in the Hand

**DOI:** 10.1055/a-2166-8995

**Published:** 2024-02-28

**Authors:** Jin Soo Kim, Chan Ju Park, Sung Hoon Koh, Dong Chul Lee, Si Young Roh, Kyung Jin Lee

**Affiliations:** 1 Department of Plastic and Reconstructive Surgery, Gwangmyeong Sungae Hospital, Gwangmyeong, Republic of Korea

**Keywords:** hand injury, wound healing, skin transplantation, hand

## Abstract

**Background** Skin defects in the hands are common injuries, and autologous skin grafting is the ideal treatment. However, complications can occur at the donor and recipient sites. This study compares the “Swing-door” technique with conventional skin grafting.

**Methods** From August 2019 to February 2023, 19 patients with skin defects of hand underwent the “Swing-door” split-thickness skin graft (STSG) technique. The thin epithelial layer was elevated with proximal part attached. Skin graft was harvested beneath. Donor site was then closed with epithelial flap like a “Swing-door”. The outcomes were evaluated in terms of healing time, scar formation, and pain at the donor and recipient sites. The data were compared with the conventional STSG.

**Results** The “Swing-door” group had lower graft take percentages, but complications did not significantly differ between the two groups. The “Swing-door” technique resulted in better cosmetic outcomes, as evidenced by lower Vancouver Scar Scale scores, faster donor site epithelialization, and reduced pain and discomfort during the early postoperative period, as measured by Visual Analog Scale.

**Conclusion** The “Swing-door” STSG is a useful alternative for treating hand skin defects.

## Introduction

Skin defects on the hands are common injuries that may result in discomfort or disability if not treated appropriately. 1 2 Split-thickness skin grafts (STSGs) are conventionally used for treating skin defects. 3 However, they can result in complications, such as delayed healing, contracture formation, and abnormal scarring at both recipient and donor sites. 4 5 Harvesting skin grafts activates dermal pain receptors and causes significant pain at the donor sites. 6

To overcome these complications, new surgical techniques, such as micrografting systems, fractional skin harvesting, and de-epithelialized skin grafts have been developed over the years in previous studies. 7 8 This study describes a technique by which a thin epithelial layer with only the proximal part still attached to the hypothenar area is harvested, followed by harvesting a partial-thickness skin flap composed of epidermal and dermal layers. The elevated epithelial layer at the donor site was covered back to the donor site and regrafted as if the swing door had closed.

The aim of this study is to compare the outcomes of “Swing-door” technique and conventional method of STSG with respect to donor site outcomes such as pain, scar, and healing time, as well as recipient site outcomes such as graft take rate, scar, and healing time.

## Methods

### Patients

**Fig. 1:**
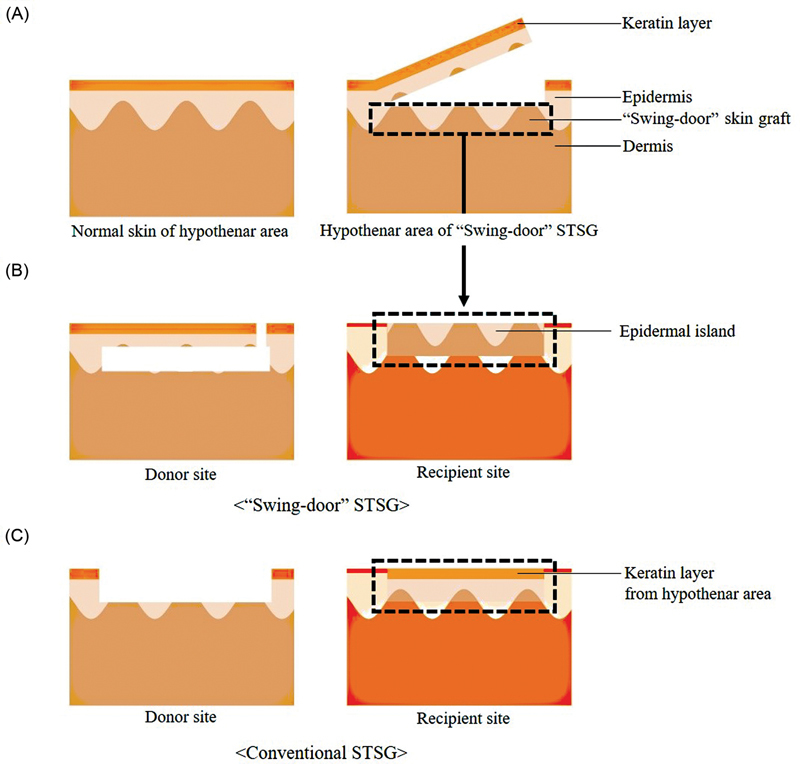
Images demonstrating ( **A** ) normal skin layers of hypothenar area and skin layers of hypothenar area during “Swing-door” harvest technique. ( **B** ) “Swing-door” STSG. ( **C** ) Conventional STSG technique in donor and recipient sites. STSG, split-thickness skin graft.

From August 2019 to February 2023, a retrospective review was conducted on patients with hand skin defects who received “Swing-door” or conventional STSG. The study included patients with skin defects greater than 1.0 × 1.0 cm, who underwent STSG using the hypothenar area ( Fig. 1 ). Data including patient demographics, underlying medical conditions, such as hypertension and diabetes mellitus, smoking history, and defect size were evaluated.

### Surgical Techniques



**Video 1** Complimentary video showing the process of “Swing-door” skin graft technique: Graft harvesting, Skin grafting and Donor closure.


**Fig. 2:**
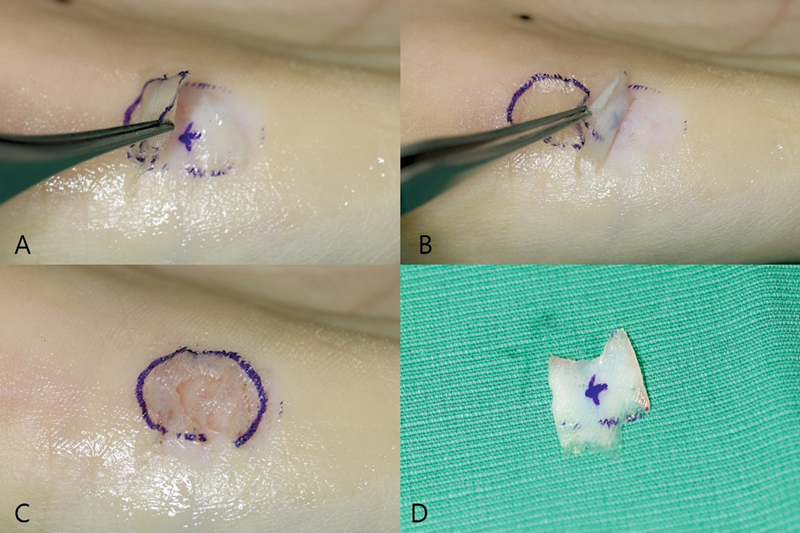
( **A** ) Epithelial flap elevation technique. It is elevated as thinly as possible with proximal side attached. ( **B** ) Harvesting of STSG beneath epithelial flap. ( **C** ) Direct donor coverage by replacing the epithelial flap like a “Swing-door.” ( **D** ) Harvested STSG (area marked with purple circle: epithelial flap; area marked with purple cross sign: STSG). STSG, split-thickness skin graft.

All surgeries were performed using a brachial plexus block. The necrotic tissue at the recipient site was completely removed. Meticulous hemostasis was achieved using an electric bipolar coagulator to prevent hematoma formation. The size of the defect was measured using a metric ruler and the skin graft was harvested from the hypothenar area. The design was made out of gentian violet and Vaseline was applied to the hypothenar surface to create a smooth and even surface. An extremely thin epithelial flap was then elevated using a razor blade with a freehand technique, taking care to elevate only the outermost epithelial layer as thinly as possible, to the extent that the letters on the razor blade could be seen through the flap. The proximal side of the flap was left attached to the donor site in a form of “Swing-door.” The skin graft, composed of epidermis and dermis, was harvested from the same area under the epithelial flap using a razor blade ( Fig. 2 ). During the harvest process, a mark was made with gentian violet to differentiate between the upper and lower layers and prevent reversal during grafting.

The skin graft was tailored to fit the size and shape of the recipient site. The graft was fixed to the recipient site by using Chromic 6–0 (AILEE Co., Busan, Korea). A tie-over dressing was applied to the skin graft after fixation, and the skin graft was evaluated after 5 days by removing the tie-over dressing. The elevated epithelial flap was placed over the recipient site. The donor site dressing was performed using Mepitel (Mölnlycke, Sweden), a wound contact layer with a silicone adhesion dressing, to maintain appropriate moisture and prevent damage to the recovery of the epithelial flap and the surrounding skin ( Video 1 ).

Patients who underwent conventional skin graft showed surgical differences in skin graft harvest and donor site management. After designing from the hypothenar area to match the size of the defect, an STSG was elevated using a razor blade. The skin graft was fixed at the donor site using Chromic 6–0. Tie-over dressing was used to fix the skin graft for 5 days. The donor site was covered with Mepitel dressing material.

### Assessment

At the recipient site, the graft take percentage was assessed on postoperative day 10. Take was evaluated based on the graft color, capillary refill time, and absence of fluid collection. Graft quality was measured using the Vancouver Scar Scale (VSS), considering vascularity, pigmentation, pliability, and height. Healing time at the recipient site was also evaluated.

At the donor site, pain levels were scored using a Visual Analog Scale (VAS) between 0 (no pain) and 10 (most painful) on postoperative day 10. Patients were followed up for at least 1 month postoperatively to evaluate the scar tissue using the VSS. Healing time at the donor site was also measured.

Two physicians scored the graft take percentage and VSS, considering donor site skin elasticity, height from the normal skin, vascularization, and pigmentation. 9 The final VSS score was calculated as the mean of both scores. The days to epithelialization were compared between the groups, with a ***p*** **-value** of less than 0.05 considered statistically significant.

### Statistical Analysis

Statistical significance between the continuous variables of the two skin graft types was analyzed using the Mann–Whitney test. Discrete variables were analyzed using Fisher's exact test. Statistical significance was set at *p* -value < 0.05. The statistical analyses were performed using the IBM SPSS Statistics (version 26.0).

## Results

Nineteen patients who underwent the “Swing-door” technique and 20 patients who underwent the conventional technique were included. In terms of wound characteristics, the mean defect size was smaller in the “Swing-door” group (1.6 cm × 1.4 cm) compared to that of the conventional group (1.8 cm × 1.5 cm). There were no statistically significant differences in incidence of complications, including infection, hematoma, and partial skin graft loss between the two groups. No patients in either group experienced total skin graft loss ( Table 1 ).

**Table 1:** Patient demographic data

	Conventional group ( *n* = 20)	“Swing-door” group ( *n* = 19)	*p* -Value
Number of patients			
Males	18	17	–
Females	2	2	–
Age, mean (y)	47.8 ± 15.2	46.1 ± 13.8	0.125
Underlying diseases			
Hypertension, *n* (%)	4 (20.0%)	3 (15.8%)	1.000
DM, *n* (%)	2 (10.0%)	2 (10.5%)	1.000
Smoking, *n* (%)	4 (20.0%)	3 (15.8%)	1.000
Mean defect size (cm)	1.8 × 1.5	1.6 × 1.4	0.291

Abbreviation: DM, diabetes mellitus.

At the recipient site, the percentage of graft take was higher in the conventional group on day 10 (95.3 vs. 96.5%, *p* -value = 0.218). However, no statistically significant correlations were observed. The “Swing-door” group showed lower VSS scores than the conventional group (4.1 ± 1.4 vs. 5.1 ± 1.6, *p* -value = 0.026). However, the time required to achieve full epithelialization was longer in the “Swing-door” group (15.1 vs. 11.7 d, *p* -value = 0.001; Table 2 ).

**Table 2:** Comparison of complications and postoperative outcomes between “Swing-door” and conventional groups in the recipient sites

	Conventional group ( *n* = 20)	“Swing-door” group ( *n* = 19)	*p* -Value
**Recipient**			
Percentage of graft take at day 10	96.5	95.3	0.218
VSS (mean, range)	5.1 ± 1.6	4.1 ± 1.4	0.026 a
Time taken to full epithelialization (mean, d)	11.7	15.1	0.001 a
Complication			
Infection, *n* (%)	0	1 (5.3)	0.401
Hematoma, *n* (%)	2 (10.0)	0	0.130
Partial skin graft loss, *n* (%)	2 (10.0)	2 (10.5)	1.000
Total skin graft loss, *n* (%)	0	0	

Abbreviation: VSS, Vancouver Scar Scale.

Note: *p* -Values were computed using Fisher's exact test to analyze the differences of each complication between the two groups.

a *p* -value <0.05 indicates statistical significance.

**Fig. 3:**
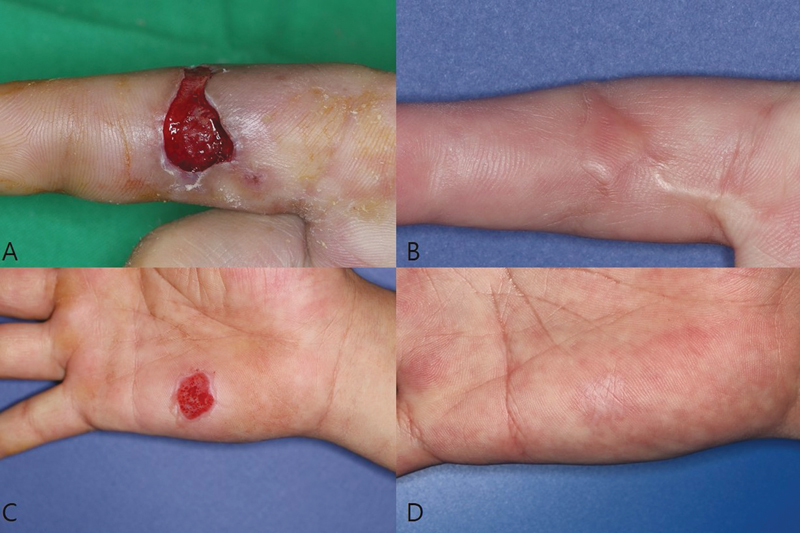
A case of conventional STSG. ( **A** ) Preoperative photograph. ( **B** ) Recipient site at postoperative 6 months. ( **C** ) Donor site at postoperative 5 days. ( **D** ) Recipient site at postoperative 6 months. STSG, split-thickness skin graft.

**Fig. 4:**
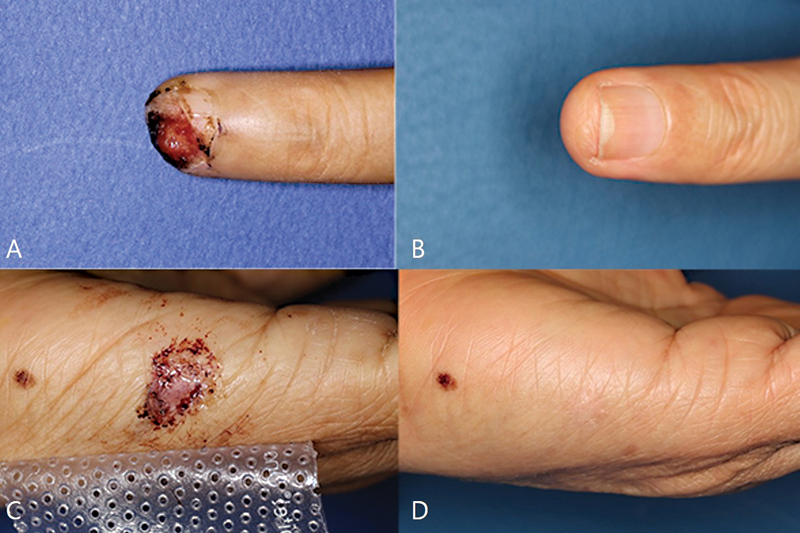
A case of “Swing-door” STSG. ( **A** ) Preoperative photograph. ( **B** ) Recipient site at postoperative 6 months. ( **C** ) Donor site at postoperative 5 days. ( **D** ) Recipient site at postoperative 6 months. STSG, split-thickness skin graft.

In the donor site, the “Swing-door” group reported significantly lower pain scores than the conventional group at postoperative days 5 and 7, with mean pain scores of 1.7 ± 0.6 versus 7.3 ± 0.2 at day 5 ( *p* -value = 0.041) and 1.5 ± 0.2 versus 6.8 ± 0.3 at day 7 ( *p* -value = 0.030). However, there was no statistically significant difference in the VAS scores between the two groups with mean pain scores of 1.3 ± 0.3 versus 5.0 ± 0.6 at day 10 ( *p* -value = 0.369). In addition, the “Swing-door” group showed significantly lower VSS scores than the conventional group (2.4 ± 0.2 vs. 3.5 ± 0.8, *p* -value = 0.048). The time taken for full epithelialization in donor site was significantly shorter in the “Swing-door” group (8.3 d) compared to the conventional group (13.5 d; Table 3 ; Figs. 3 and 4 ).

**Table 3:** Comparison of postoperative outcomes between “Swing-door” and conventional groups in the donor sites

	Conventional group ( *n* = 20)	“Swing-door” group ( *n* = 19)	*p* -Value
**Donor**			
VAS at day 5 (mean, range)	7.3 ± 0.2	1.7 ± 0.6	0.041 a
VAS at day 7 (mean, range)	6.8 ± 0.3	1.5 ± 0.2	0.030 a
VAS at day 10 (mean, range)	5.0 ± 0.6	1.3 ± 0.3	0.369
VSS (mean, range)	3.5 ± 0.8	2.4 ± 0.2	0.048 a
Time taken to full epithelialization (mean, d)	13.5	8.3	0.005 a

Abbreviations: VAS, visual analog scale; VSS, Vancouver Scar Scale.

a *p* -value <0.05 indicates statistical significance.

## Discussion

The STSG is a useful reconstructive option for skin defects. 10 However, patients often experience complications, such as pain, pruritus, hypertrophic scarring, color mismatch, dryness, and contracture. 11 12 Modified surgical techniques, like the “Swing-door” method, have been developed to overcome these complications. Our comparative study suggests that the “Swing-door” technique has several advantages over the conventional method.

**Fig. 5:**
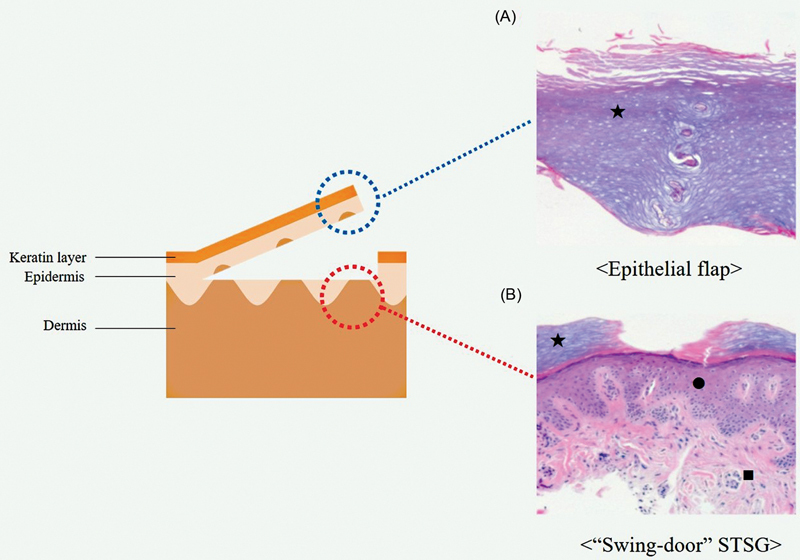
Histological comparison between “Swing-door” and conventional STSG. ( **A** ) Epithelial flap mostly consisted of keratinized layer of stratum corneum (black star) and partial epidermal layers including stratum granulosum and spinosum. ( **B** ) Keratinized layer of stratum corneum (black star) was mostly removed from “Swing-door” skin graft. Epidermal layer including stratum basale (black circle) and dermis (black square) were preserved (H&E, ×100). STSG, split-thickness skin graft.

At the recipient sites, the “Swing-door” group showed better outcomes on the VSS. The VSS score assesses scars based on various parameters, such as pigmentation, pliability, and thickness, with higher scores indicating worse outcomes. The lower VSS score in the “Swing-door” group indicates that this technique resulted in better cosmetic outcomes compared to the conventional technique at the recipient sites. In order to interpret this result, specimens from the epithelial flap and “Swing-door” skin graft were collected for histological evaluation. According to the results, when harvested using the “Swing-door” technique, the epithelium on the surface of the skin graft was partially removed, leaving the epidermis in the form of epithelial islands on the dermis ( Fig. 5B ). The partially remaining epithelial islands are hypothesized to undergo reepithelialization and healing through cellular multiplication and migration toward the peripheral boundaries. 13 This process is believed to result in less color mismatch and step differences compared to conventional skin grafting.

The scar quality appears to be associated with the keratin layer. According to a previous study, increased expression of keratin K2e in the upper spinous and granular layers of the interfollicular epidermis, such as in keloid scars, is highly associated with scar formation, such as keloid scars. 14 There appears to be a relationship between the number of keratin layers and scar quality. Histological studies indicate that the keratin layer of the “Swing-door” skin graft is mostly removed, which may be the reason for the superior pliability measured by the VSS in the “Swing-door” group.

The “Swing-door” group showed slower healing time and lower take rate in the recipient site. Wound healing is thought to occur simultaneously in the two different areas. While the skin graft enters the wound bed on the inner side, the de-epithelialized skin graft on the outer side requires reepithelialization to occur simultaneously. However, the most critical part of skin graft take is known to be revascularization in recipient bed which requires factors such as VEGF and HIF-1a that are known to be responsible for the neoangiogenesis process. 15 The graft take is heavily dependent on the recipient site. Therefore, the slower healing time and lower take rate of the “Swing-door” group in the recipient site may not be caused by technical difference between “Swing-door” and conventional methods, but rather due to differences in conditions of the recipient sites.

Hematoma is one of the main complications associated with graft failure, as it acts as a barrier between the wound bed and skin grafts, which ultimately leads to skin graft failure. 10 The hematoma that occurred in this study was discovered after the removal of the tie-over dressing. After creating a hole with an 18-gauge needle for hematoma removal, they were resolved without any graft loss. Skin grafts can also be lost due to infections that occur during the tie-over period or in the early weeks postoperation, even after a successful graft take. 16 One case of infection that occurred in our study showed sign of infection with partial melting down of the graft at the time of removal of tie-over dressing. This led to partial skin graft loss, but naturally underwent full reepithelialization without the need for additional surgery. The incidence of complications, including hematoma and infection, was similar in both groups at the recipient site and did not result in detrimental outcome which may have caused additional surgery, indicating that the “Swing-door” technique does not significantly increase the incidence and severity of complications compared to the conventional method.

In the “Swing-door” group, histologic study has shown that the epithelial flap contains some epithelial cells on the inner side ( Fig. 5A ). By replacing the epithelial flap with the donor site, the flap functioned similarly to a very thin graft. This epithelial regraft, which was previously described as a tissue with independent reepithelialization potential, 17 assists in the healing process. Simultaneously, it acts as a partial biological dressing, which appears to enable faster epithelialization at the donor site. In the donor site, the healing time was faster in the “Swing-door” group. A previous study reported that the use of occlusive or semiocclusive dressings that maintain a moist environment results in earlier completion of epithelialization compared to dry and open dressings. 18 Furthermore, they offer optimal protection against wound dehydration, contamination, and mechanical trauma, thereby promoting rapid healing. 19

Donor site pain is one of the most burdensome concerns for patients. The “Swing-door” technique was associated with significantly less pain and discomfort around the donor site during the early postoperative period, as reflected by the lower VAS score at postoperative days 5 and 7. At the donor site of an STSG, the injured nerve endings exposed to external stimuli cause heightened pain compared to the recipient site. The use of an occlusive environment reduces pain by protecting nerve endings from oxygen exposure and decreasing the concentration of macrophage-derived arachidonic acid metabolites that can exacerbate pain. 20 Since the epithelial flap is instantly replaced back to its donor site intraoperatively, the time nerve endings are exposed to outer environment is significantly decreased in “Swing-door” technique, thus leading to less pain for the patients. This finding is particularly important for improving patient satisfaction and compliance with postoperative care, as pain and discomfort can significantly affect the quality of life and recovery process. 21

The “Swing-door” technique also showed superior cosmetic outcomes compared to the conventional technique, as evidenced by the lower VSS score in the donor site. According to the histological study by Thompson, 22 regrafting of the donor site of STSG promotes advanced regeneration of elastic tissue, thus resulting in less hypertrophic scarring and improved cosmetic results. This was confirmed in the present study. In the “Swing-door” technique, it is believed that the thin epithelial flap acted as a regraft by being directly folded back onto the donor site and contributed to less hypertrophic scar.

The potential advantage of the “Swing-door” technique includes cost savings in postoperative management. Since the epithelial flap is directly replaced at the donor site, it partially acts as a biological dressing, thus promoting wound healing without the need for additional dressing material. This technique is expected to reduce expenses associated with donor site dressing materials.

One limitation of our study is the potential variation in the thickness of the skin graft harvested when using the freehand technique, as the dermatomes were not used. This could have affected the results, as studies have reported that thicker skin grafts can affect the take and healing. 23 To minimize this variability, our study was conducted using cases performed by only one surgeon. However, the use of more standardized operative tools or dermatome devices could overcome this limitation.

Another limitation is that this technique is only applicable to the hypothenar and plantar areas, where the keratin layers are thicker than those in other parts of the body. The hypothenar region is one of the most glabrous and thick areas of the human body. Studies have shown that keratinocytes within the palm and sole epidermis possess exceptionally large amounts of keratin filaments in their cytoplasm, unlike other parts of the body with thin skin. 24 This may be due to the expression of palmoplantar-specific keratin in the hypothenar and plantar arch areas. 25 Therefore, there is a difficulty in that the size of the skin defect is limited to the size of the skin graft that can be harvested from the hypothenar area. Furthermore, since hypothenar skin has less melanin-containing pigment cells and compact connective tissues, it serves as an optimal candidate for the coverage of skin defects in hands. 26 Therefore, the “Swing-door” technique has a limitation in that it can mainly be used for small skin defects on the hand.

Moreover, since the percentage of skin graft take and VSS were subjectively determined by the observers, biases may have been introduced. To address this issue, two different physicians evaluated the outcomes. However, the development of more objective evaluation tools in the future could overcome this limitation.

It is important to note that both the “Swing-door” group and the conventional group had relatively low case numbers in this study. The limited sample size may have influenced the statistical power of our analysis. Therefore, our results should be interpreted with caution. Future studies with larger sample sizes may provide further insight into the differences between these techniques.

The “Swing-door” technique for split-thickness skin grafting in the treatment of skin defects of the hands showed satisfiable results. This technique offers advantages over conventional techniques in terms of pain, faster wound healing, improved cosmetic outcomes at the donor site, and comparable outcomes at the recipient site.
